# Characterization and Etiopathogenic Approach of Pediatric Renal Biopsy Patients in a Colombian Medical Center from 2007-2017

**DOI:** 10.1155/2018/9603453

**Published:** 2018-06-28

**Authors:** Mayerly Prada Rico, Carmen Inés Rodríguez Cuellar, Monica Fernandez Hernandez, Luz Stella González Chaparro, Olga Lucía Prado Agredo, Ricardo Gastelbondo Amaya

**Affiliations:** ^1^Pediatric Nephrology Division, Pediatrics Department, Fundación Cardioinfantil, Bogotá, Cundinamarca, Colombia; ^2^Pediatric Emergency Care Division, Pediatrics Department, Fundación Cardioinfantil, Bogotá, Cundinamarca, Colombia; ^3^Pediatric Endocrinology Division, Pediatrics Department, Fundación Cardioinfantil, Bogotá, Cundinamarca, Colombia

## Abstract

**Introduction:**

Renal biopsy is the principal instrument to evaluate the diagnosis and prognosis of children with kidney disease. There are relatively few studies establishing epidemiology of its findings in the pediatric population.

**Methods:**

A descriptive study was conducted to describe characteristics of pediatric patients who had undergone a renal biopsy over the last 10 years in a national reference center, trying to accomplish an etiopathogenic approach of biopsy findings.

**Results:**

241 patients were included. Most frequent indications were nephrotic syndrome (34.1%) and systemic disease with renal involvement (30.2%). The most prevalent biopsy diagnosis was glomerulonephritis (44%) and among these patients, glomerulonephritis mediated by immune complexes was the most frequent pathogenic type (90.5%). When the biopsy was indicated for proteinuria plus hematuria and systemic disease with renal involvement, the most frequent biopsy diagnosis was glomerulonephritis (60 and 85%, respectively). For isolated hematuria, the predominant biopsy diagnosis was inherited diseases of the glomerular basement membrane (70%) and for nephrotic syndrome, podocytopathy (82%). Glomerulonephritis was more frequent in patients older than 10 yrs (65%) and the rate of postbiopsy major complications was low (1.2%).

**Conclusion:**

Immune complex glomerulonephritis was the most frequent histological finding, differing from previous reports. To our knowledge this is the first description that classifies biopsy findings according to the probable pathogenic mechanism.

## 1. Introduction

 Percutaneous renal biopsy allows direct study of renal pathology [1]. Although many pediatric patients with kidney diseases can be properly diagnosed without it, in some situations biopsies are not only a diagnostic resource. Findings also determine prognosis and guide treatments [2, 3]. In order to establish an adequate diagnostic and therapeutic approach to kidney disease based on biopsy histological findings, it is essential to recognize the pathogenic mechanisms involved, improving the understanding of kidney disease [2]. Mayo Clinic 2015 Nephrologists and Renal Pathologists Consensus is an example of a diagnostic and classification system of glomerulonephritis based on etiology and pathogenesis [4].

Since the first renal biopsy description in 1934 [5], different series have been published describing the indications, histopathological findings, and complications in children [2, 6–23]. However, an etiopathogenic classification of biopsy findings has not been performed to date. Herein, we attempt to accomplish such an approach.

## 2. Materials and Methods

Retrospective review of clinical and pathology reports of under 18 yrs patients who underwent renal biopsy between January 2007 and May 2017 at Fundación Cardioinfantil, Bogota, Colombia. Demographics, clinical variables, indication of renal biopsy, histological findings, and biopsy complications were analyzed.

Renal biopsy indications were nephrotic syndrome, systemic disease with renal involvement, isolated subnephrotic proteinuria, proteinuria and hematuria, isolated hematuria, glomerular filtration rate impairment without a known cause, isolated nephrotic proteinuria, and nephritic syndrome. Isolated subnephrotic proteinuria was defined as the presence of 4 to 40 mg/m^2^/hour in a 24-hour urine collection or a urinary protein/creatinine ratio between 0.2 and 2 in an isolated sample without other symptoms (edema, dyslipidemia, hypoalbuminemia, hematuria, etc.). Isolated hematuria was defined as the presence of macro or microscopic hematuria without other symptoms (proteinuria, edema, hypertension, decreased glomerular filtration rate, etc.).

Histological findings were classified as detailed in Table 1. Biopsy complications were classified as follows: (1) major: if they involved additional interventions or outcomes (resuscitation, transfusion of blood components, surgery, prolonged hospital stay, readmission, death, infection, or macroscopic hematuria); (2) minors: if they did not involve additional interventions or outcomes.

Patients were admitted on the previous biopsy day and underwent a clinical evaluation including: clinical history, physical examination, and complementary bloodwork (complete blood count, partial thromboplastin time, and prothrombin time). Postbiopsy evaluation was performed as follows: (1) Complete blood count 6 hours after the intervention or earlier if required (hemodynamic instability, macroscopic hematuria, etc.). (2) Hourly vital signs and macroscopic hematuria surveillance over the first 6 to 12 hours. (3) Kidney ultrasound taken after 24 hours.

All the biopsies were ultrasound guided and performed by interventional radiologists under general anesthesia. An automatic percutaneous biopsy instrument and 18G needles were used for all patients regardless of their stature.

Samples were studied under light microscopy, immunofluorescence, and electron microscopy.

### 2.1. Statistical Analysis

Description of demographics, clinical findings, and relevant variables related to the histopathological findings, through relative frequency measurements. Comparative tables and graphs with frequency distribution.

All analyses were performed using STATA version 9 software (StataCorp LP, College Station, TX).

## 3. Results

Total number of patients that were included in biopsy was 241; 58% (140/241) females, 78.8% (190/241) original from Colombia's central region. Renal biopsy mean age was 11 yrs (4.3 SDS).

27.8% (67/241) had impaired glomerular filtration rate and 80.4% (194/241) comorbidities such as acute kidney injury (13.7%; 33/241), systemic diseases (29.8%; 72/241), coagulopathy (2.9%; 7/241), high blood pressure (32.7%; 79/241), and solitary kidney (1.6%; 4/241).  Table 2 shows kidney disease stage and the associated systemic disease.

### 3.1. Renal Biopsy Indications

**Figure 1:**
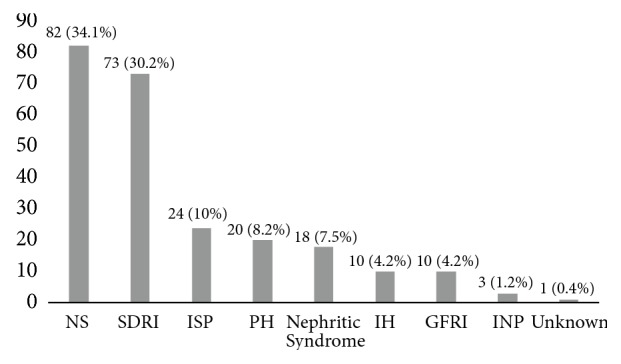
Renal biopsy indications. NS: nephrotic syndrome; SDRI: Systemic disease with renal involvement; ISP: isolated subnephrotic proteinuria; PH: proteinuria and hematuria; IH: isolated hematuria; GFRI: glomerular filtration rate impairment without a known cause; INP: isolated nephrotic proteinuria.

Renal biopsy indications are illustrated in Figure 1. Among nephrotic syndrome patients 46.3% (38/82) had steroid-resistant nephrotic syndrome (SRNS), 44% (36/82) steroid-dependent nephrotic syndrome (SDNS), and 9.7% (8/82) frequently relapsing nephrotic syndrome (FRNS). Indications frequency according to age is shown in Table 3. Systemic disease with renal involvement was the most frequent indication in children older than 10 yrs while nephrotic syndrome was the most frequent indication in children younger than 10 yrs. Other indications showed a variable distribution between the groups.

### 3.2. Histopathological Findings

The most frequent biopsy diagnosis was glomerulonephritis (44%), followed by podocytopathy (33.6%), basement membrane disease (5.3%), and tubulointerstitial disease (5.8%).

Among glomerulonephritis (106/241) the most common pathogenic type was glomerulonephritis mediated by immune complexes (90.5%; 96/106) and of this, the most common specific entity was lupus nephritis (50%; 48/96).

In every podocytopathy cases the pathogenic type was primary nephrotic syndrome and of this, the most common specific entity was minimal changes disease (MCD) (58%; 47/81) (Table 4).

5 out of 48 (10.4%) lupus nephritis patients were classified as class I or II, 37/48 (77%) as class III or IV, 4/48 (8.3%) as class V, and 1/48 (2%) as class VI.

Regarding the IgA Nephropathy subgroup of patients, 31.4% (11/35) had a previous medical history of systemic diseases. 72.7% (8/11) had Schönlein Henoch purpura, 18.1% (2/11) immune thrombocytopenic purpura, and 9.0% (1/11) erythematous systemic lupus. The most frequent Oxford classification was M0S0E0T0 in 28.5% (10/35) followed by M1S0E1T0 in 14.2% (5/35) and M1S1E1T0 in 14.2% (5/35).

Among 28 patients with focal segmental glomerulosclerosis (FSGS) the histopathological variants found were as follows: not-otherwise-specified in 42.8% (12/28), cellular 39.2% (11/28), collapsing disease 7.1% (2/28), tip 7.1% (2/28), and perihilar 3.5% (1/28).

### 3.3. Renal Biopsy Indications and Histopathological Findings Correlation

**Figure 2:**
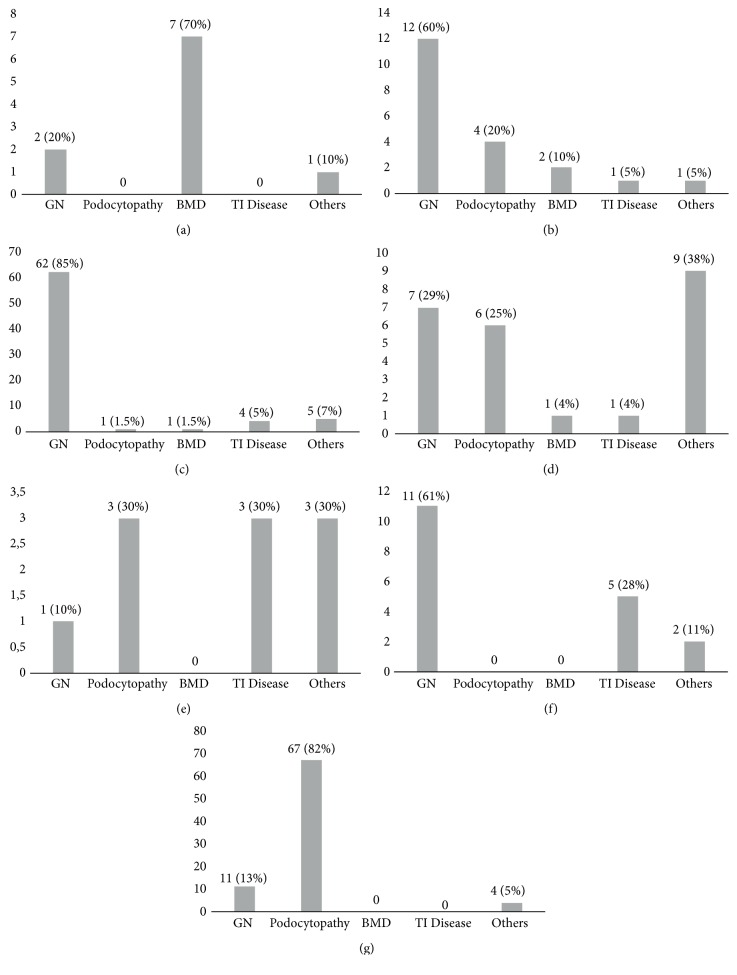
Renal biopsy indications and biopsy diagnoses. (a) Isolated hematuria; (b) proteinuria and hematuria; (c) systemic disease with renal involvement; (d) isolated subnephrotic proteinuria; (e) glomerular filtration rate impairment without a known cause; (f) nephritic syndrome; (g) nephrotic syndrome; GN: glomerulonephritis; TI: tubulointerstitial; BMD: basement membrane disease.

In the subgroup of patients undergoing renal biopsy due to proteinuria and hematuria, systemic disease with renal involvement, and nephritic syndrome, the most frequent biopsy diagnosis was glomerulonephritis that was present in more than 50% of the cases. Additionally, when the indication was isolated hematuria and nephrotic syndrome, the most common biopsy diagnosis was basement membrane disease and podocytopathy, respectively. On the contrary, for glomerular filtration rate impairment without a known cause and isolated subnephrotic proteinuria patients, the distribution of biopsy diagnoses was variable (Figure 2).

Glomerulonephritis was more frequent in patients with SRNS versus SDNS/FRNS (24% and 4%, respectively) and in patients with proteinuria plus hematuria versus isolated hematuria (60% and 20%, respectively).

**Figure 3:**
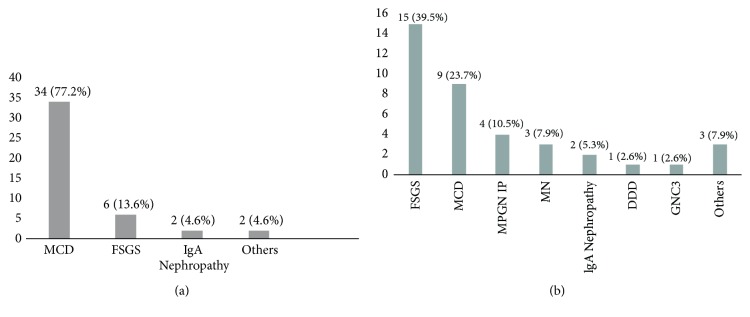
Specific entity according to corticosteroids response. (a) Steroid-dependent or frequently relapsing nephrotic syndrome; (b) steroid-resistant nephrotic syndrome; MCD: minimal change disease; FSGS: focal segmental glomerulosclerosis; MPGN IP: membranoproliferative glomerulonephritis immunoglobulin positive; MN: membranous nephropathy; DDD: dense deposit disease; C3GN: C3 glomerulonephritis.

When renal biopsy indication was nephrotic syndrome (n=82), the most prevalent specific entity was MCD (52.4%; 43/82), followed by FSGS (25%; 21/82). However, when analyzing according to corticosteroids response, the FSGS was more frequent in SRNS patients (39.5%; 15/38) while MCD in SDNS/FRNS (77.2%; 34/44) (Figure 3).

In 7/10 isolated hematuria cases, the specific entity was thin basement membrane disease, in 2/10 IgA Nephropathy, and in one case the findings were nonspecific.

**Figure 4:**
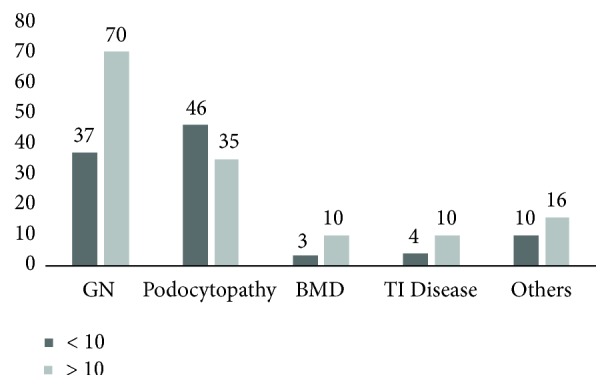
Biopsy diagnosis according to age group. GN: glomerulonephritis; TI: tubulointerstitial; BMD: basement membrane disease.

As far as biopsy diagnosis according to age group, glomerulonephritis was more frequent in children older than 10 yrs than in children under 10 yrs (Figure 4).

### 3.4. Complications

The only postbiopsy complication was subcapsular hematoma, present in 56 out of 241 cases (23.2%). In 54/56 cases (98.8%) hematomas were < 2 cm and there was no need of additional interventions. Due to their size, hemodynamic instability, anemia, and transfusion need, 2/56 cases (1.2%) were considered as a major complication. There were no cases of perforation, infection, fistula, or death.

## 4. Discussion

Over the years there is growing interest in recognizing the significance of histomorphological biopsy findings and pathogenic mechanisms involved in diseases affecting glomerular, vascular, or tubulointerstitial kidney structures. Thus, terms such as podocytopathy arise to group those entities in which podocyte biology is affected, as FSGS or MCD [24]. Likewise, membranoproliferative glomerulonephritis classification has been reconsidered, taking into account not only histomorphological findings, but also the presence of immune complex or complement as a pathogenic mechanism [25, 26]. These new approaches help to understand histopathological lesions, identify new or under diagnosed entities, and establish therapeutic guidelines and further investigations needed [26].

Based on this need, the Mayo Clinic/Renal Pathology Society Consensus Report on Pathologic Classification, Diagnosis, and Reporting of Glomerulonephritis emerged in 2015. This approach includes a primary diagnosis constituted by the pathogenic type, specific entity, injury pattern, and the lesion classification according to the case, followed by a secondary diagnosis, reporting coexisting injuries not necessarily associated with the primary diagnosis [4].

To our knowledge, there are relatively few studies describing renal biopsy indications, findings, and complications epidemiology in pediatrics (Table 5). None of them have a pathogenic approach of biopsy histological findings being this, the first pediatric description trying to achieve it.

Similar to previous pediatric reports, renal biopsy mean age in our study was 9 to 11 years [6, 8–10, 13, 18, 20, 22, 23] and nephrotic syndrome was the most frequent indication [2, 6–15, 18, 20, 21]. However, unlike other studies [8, 13, 22] there was a high proportion of patients with systemic disease and the prevalence of isolated hematuria was low.

Past descriptions showed “primary glomerular diseases” as the most frequent biopsy finding, including entities like MCD, FSGS, IgA Nephropathy, among others [2, 6, 8–17]. Opposite to this, in our study, glomerulonephritis was the most common finding. Considering immune complex, complement, and other immunological mechanisms involved in this entity, the presence of systemic diseases in a significant proportion of the children included seems to be like a plausible explanation (Table 2). However, it is also possible that a pathophysiological approach of biopsy findings made us categorize immune complex glomerulonephritis entities that are traditionally considered as primary.

The finding of lupus nephritis as the most common specific entity among immune complexes mediated glomerulonephritis is striking. Considering that in previous series the most frequent specific entity was IgA Nephropathy [13, 21, 27]. This may suggest a different epidemiological behavior in our population.

Due to insufficient data in medical records, classification of tubulointerstitial diseases according to pathogenic mechanisms was not possible. This aspect should be taken into account in future descriptions.

This study ratifies variability in glomerulonephritis clinical manifestations [28, 29] but shows that glomerulonephritis mediated by immune mechanisms as a finding could be more likely in children with proteinuria and hematuria, systemic disease with renal involvement, nephritic syndrome, and SRNS or being older than 10 yrs, unlike podocytopathies, which were more prevalent in children with SDNS/FRNS and younger than 10 yrs.

In recent years emphasis has been placed on nephrotic syndrome epidemiology changes, due to an increase in FSGS lesions [30, 31]. But, in our series, MCD was the most common histopathological lesion. Nonetheless, when analyzing by subgroups according to corticosteroids response, FSGS was more frequent in SRNS while MCD was more frequent in SDNS/FRNS.

The results endorse clinical-pathological correlation relevance, considering agreement in some prebiopsy indications and postbiopsy findings.

Postrenal biopsy complications frequency is variable according to the series previously published (Table 5). In our study, the only complication was subcapsular hematoma in 23.2% of the cases. Nevertheless, only 1.2% (2 cases) was considered as a major complication. These two cases corresponded to patients admitted to the pediatric intensive care unit due to systemic lupus erythematous debut associated with acute kidney injury and dialysis requirement. Factors as uremia, arterial hypertension, and autoimmune disease may have contributed to this complication development. In the present study, major complications prevalence is much lower compared to other reports 12 to 30.8% of the cases [18, 32, 33]. This allows us to conclude that, in our center, percutaneous renal biopsy is a safe procedure with a major complications prevalence of less than 5%, concordant to previously established standards [32].

## 5. Conclusion

In children, classification of histopathological findings in renal biopsy based on the probable etiopathogenic mechanisms constitutes a key instrument not only for an adequate diagnostic and therapeutic approach, but also for the understanding of renal disease epidemiological behavior, according to the population.

## Figures and Tables

**Table 1:** Histopathological findings classification.

**Category**	**Pathogenic type**	**Specific disease entity**	**Scores or class**
**Glomerulonephritis (GN)**	Immune-complex GN	LN, IgA Nephropathy, MPGN Immunoglobulin positive, Post infectious acute GN	Oxford/MEST score for IgA Nephropathy, ISN/RPS score for NL
	Complement Mediated GN	aHUS, C3GN, DDD	
	Pauci-immune GN	MPO-ANCA GN	
**Podocytopathy **	Primary NS	MCD, FSGS, MN	Not-otherwise-specified, cellular, collapsing disease, tip and perihilar variants for FSGS
**Basement membrane disease (BMD)**	Inherited Diseases of the Glomerular Basement Membrane	TMB, AS	
**Tubulointerstitial disease **	Acute TIN Chronic TIN		
**Others**	Nonspecific findings Normal End stage kidney disease		

LN: Lupus Nephritis; MPGN: Membranoproliferative Glomerulonephritis; aHUS: Atypical Hemolytic Uremic Syndrome; C3GN: C3 Glomerulonephritis; DDD: Dense Deposit Disease; MPO: Myeloperoxidase; ANCA: Anti-neutrophil Cytoplasmic Antibodies; NS: Nephrotic Syndrome; MCD: Minimal Change Disease; FSGS: Focal Segmental Glomerulosclerosis; MN: Membranous Nephropathy; TMB: Thin Membrane Disease; AS: Alport's Syndrome; TIN: Tubulointerstitial Nephritis; MEST: mesangial hypercellularity (M), segmental sclerosis (S), interstitial fibrosis/tubular atrophy (T) lesions; ISN/RPS: International Society of Nephrology/ Renal Pathology Society

**Table 2:** Kidney disease stage and associated systemic disease.

		**n (**%**)**
**Kidney disease stage according to KDIGO classification**	G1	194 (80.5)
	G2	15 (6.2)
	G3A	5 (2.1)
	G3B	3 (1.3)
	G4	15 (6.2)
	G5	6 (2.5)
	Unknown	3 (1.2)
**Systemic diseases**	ESL	51 (21.2)
	SHP	11 (4.5)
	ITP	4 (1.7)
	Others	6 (2.4)

ESL: erythematosus systemic lupus; SHP: Schönlein-Henoch purpura; ITP: immune thrombocytopenic purpura.

**Table 3:** Indications frequency according to age.

**Indication**	**<10 years**	**>10 years**
	n (%)	n (%)
**NS**	48 (48%)	34 (24.1%)
**SDRI**	16 (16%)	57 (40.5%)
**ISP**	9 (9%)	15 (10.6%)
**PH**	3 (3%)	17 (12%)
**Nephritic Syndrome**	11 (11%)	7 (5%)
**IH**	6 (6%)	4 (2.83%)
**GFRI**	4 (4%)	6 (4.3%)
**INP**	2 (2%)	1 (0.7%)
**Unknown**	1 (1%)	0
**Total**	100 (100%)	141 (100%)

NS: nephrotic syndrome; SDRI: systemic disease with renal involvement; ISP: isolated subnephrotic proteinuria; PH: proteinuria and hematuria; IH: isolated hematuria; GFRI: glomerular filtration rate impairment without a known cause; INP: isolated nephrotic proteinuria.

**Table 4:** Distribution of histopathological findings.

**Histopathological findings**	**n (**%**)**
**1. Glomerulonephritis (GN)**	**106 (44)**

1.1 Immune-complex GN	96 (39.8)
LN	48 (19.9)
IgA Nephropathy	35 (14.5)
MPGN Immunoglobulin positive	8 (3.3)
Post infectious acute GN	5 (2)
1.2 Complement Mediated GN	9 (3.7)
aHUS	4 (1.6)
C3GN	3 (1.2)
DDD	2 (0.8)
1.3 Pauci-immune GN	1 (0.41)
MPO-ANCA GN	1 (0.41)

**2. Podocytopathy **	**81 (33.6)**

2.1 Primary NS	81 (33.6)
MCD	47 (19.5)
FSGS	28 (11.6)
MN	6 (2.48)

**3. Basement membrane disease**	**13 (5.3)**

3.1 Inherited diseases of the glomerular basement membrane	
TMB	13 (5.3)

**4. Tubulointerstitial disease **	**14 (5.8)**

Acute TIN	14 (5.8)

**5. Others**	**27 (11.2)**

Nonspecific findings	18 (7.4)
Normal	4 (1.6)
End Stage kidney	5 (2)

LN: lupus nephritis; MPGN: membranoproliferative glomerulonephritis; aHUS: atypical hemolytic uremic syndrome; C3GN: C3 glomerulonephritis; DDD: dense deposit disease; MPO: myeloperoxidase; ANCA: anti-neutrophil cytoplasmic antibodies; NS: nephrotic syndrome; MCD: minimal change disease; FSGS: focal segmental glomerulosclerosis; MN: membranous nephropathy; TMB: thin membrane disease; TIN: tubulointerstitial nephritis.

**Table 5:** Selected studies on the renal biopsy in children.

**Autor, year**	**Country**	**n**	**Ages, mean age ** **years**	**Most frequent indication (**%**)**	**Most frequent postbiopsy diagnosis (**%**)**	**Most frequent specific disease entity (**%**)**	**Complications (%)**
**Coppo R, 1998**	Italy	432	< 15 8.96±3.7	SP (31.2)	Glomerular diseases (66.9)	IgAGN (18.8)	ND

**Madani A, 2003**	Iran	601	0-16	ND	ND	MCD (18.5)	ND

**Bazina M, 2007 **	Croacia	65	1 - 18 11.1±4.8	NS (41.5)	ND	MesPGN (27.7)	Retroperitoneal hemorrhage which needed blood transfusion (3.07)

**Yuen L, 2008**	China	171	2- 24 11	Native kidneys: SDRI (34)	Native kidneys: Glomerular diseases (66)	MCD (14)	ND

**Piotto GH, 2008**	Brazil	262	9.8 ± 4.2	NS (42.4)	ND	MCD (61.3)	Gross hematuria (5.6), bradycardia and hypotension (0.7)

**Demircin G, 2009**	Turkey	614	0.1 - 24 10.4	NS (47)	Glomerular diseases (61.2)	MPGN (11.1)	Perirenal hematoma (12.4), gross hematuria (2.6), AV fistula (1)

**Orta N, 2009**	Venezuela	395	0.2 – 20 11 ± 7.2	NS (55)	Glomerular diseases (77)	MCD (46)	Transient hematuria (5), perirenal hematoma (<1), gut perforation (<0.2), bleeding which required blood transfusion (<0.5), nephrectomy because of incontrollable bleeding (0.2)

**Lanewala,2009**	Pakistan	801	0.4 -18 10.59 ± 4.54	Native Kidneys: NS (69.4)	Glomerular diseases (87.64)	MCD (29.4)	ND

**Absar A, 2010**	Pakistán	40	1 - 14 9	NS (61)	ND	MCD (37)	ND

**Miller M, 2010**	Jamaica	157	0.1 - 11 7.58	NS (57.4)	Glomerular diseases (53)	DPGN (27.7)	ND

**Abdelraheem MB, 2010**	Sudan	321	0.2 - 16 8.71	NS (62.9)	ND	MCD (29.9)	ND

**Cots JV, 2010**	Spain	164	0.6 - 18	SDNS (16.1)	ND	IgAGN (26.1)	Perirrenal hematoma (72.2), gross Hematuria (6), renal hematoma and hypotension (0.6)

**Printza, 2011**	Greece	81	1-18 9.56	NS (34.5)	Glomerular diseases (69)	FSGS (15)	Subcapsular hematoma (11)

**Abdullah LS, 2012**	Arabia Saudí	252	0- 17 11.2	NS (48.3)	Glomerular diseases (88.4)	MesPGN (19.8)	ND

**Paripovic D, 2012**	Serbia	150	0.2 - 20 11.5	NS (32.9)	Glomerular diseases (57.4)	FSGS (20.9)	ND

**Bakr A, 2014**	Egypt	1096	0.2 - 18 9.2 +/- 3.7	SRNS (28.4)	Glomerular diseases (67.4)	MCD (21.8)	ND

**Hadidi R, 2014**	Jordan	55	1 - 13	SRNS (25)	ND	MCD (27)	Gross hematuria (5.5)

**Mutalik P, 2015**	India	140	0.6 - 14 6.31 ± 3.75	NS (77.9)	ND	MCD (33.5)	ND

**Imtiaz S, 2016**	Pakistan	423	10.48 +/- 4.58	NS (74.2)	Glomerular diseases (85.1)	MCD (30.3)	ND

**Abdel-Hafez, 2017**	Egypt	210	0.3 - 18 10.51 ± 3.81	NS (43.89)	ND	MCD (22.17)	Local pain (60.58), gross hematuria (5.88), perirrenal hematoma (1.36)

SP: subnephrotic proteinuria; NS: nephrotic syndrome; SDRI: systemic disease with renal involvement; IgAGN: IgA nephropathy; MCD: minimal change disease; FSGS: focal segmental glomerulosclerosis, MPGN: membranoproliferative glomerulonephritis; DPGN: diffuse proliferative glomerulonephritis; SRNS: steroid-resistant nephrotic syndrome; MesPGN: mesangioproliferative glomerulonephritis; SDNS: steroid-dependent nephrotic syndrome; ND: not described; AV: arteriovenous.

## Data Availability

The data used to support the findings of this study are property of Fundación Cardioinfantil. Access to these data will be considered by the author upon request, with permission of Research Department and Institutional Ethics Board Committee.
